# Cognitive outcomes after extracranial-intracranial bypass surgery in elderly patients diagnosed with atherosclerotic cerebral steno-occlusive artery disease

**DOI:** 10.3389/fnagi.2025.1548319

**Published:** 2025-03-05

**Authors:** Yu Duan, Jian Li, Xin Zhang, Shihong Li, Qiliang Chai, Yingying Zhang, Guohui Huang, Ziwei Xu, Zhuyu Li, Renling Mao, Dongwei Dai

**Affiliations:** ^1^Department of Neurosurgery, Huadong Hospital, Fudan University, Shanghai, China; ^2^Department of Neurosurgery, Huashan Hospital, Fudan University, Shanghai, China; ^3^Department of Imaging, Huadong Hospital, Fudan University, Shanghai, China; ^4^Department of Medical Ultrasonics, Huadong Hospital, Fudan University, Shanghai, China; ^5^Department of Neurology, Zhongshan Hospital, Fudan University, Shanghai, China; ^6^Department of Neurology, Huadong Hospital, Fudan University, Shanghai, China

**Keywords:** elderly, arterial steno-occlusive disease, bypass surgery, cognitive function, cerebral perfusion

## Abstract

**Background:**

The safety and clinical effectiveness of extracranial–intracranial (EC–IC) bypass surgery in elderly patients with atherosclerotic internal carotid artery and/or middle cerebral artery steno-occlusive (ACMSO) disease remain ambiguous. Here, we analyzed our experience of EC-IC bypass surgery to evaluate its clinical safety and effect on the cognitive function for elderly patients with ACMSO.

**Methods:**

This retrospective study enrolled patients >60 years of age diagnosed with ACMSO who underwent EC–IC bypass surgery at the authors' center between January 2018 and January 2021. Indications for bypass surgery included symptomatic ACMSO defined by cerebral angiography and evidence of relative hypoperfusion in the territories of steno-occlusive arteries based on computed tomography perfusion (CTP) neuroimaging. All patients underwent the Montreal Cognitive Assessment preoperatively and 2 years after bypass surgery. Clinical data, such as the National Institute of Health Stroke Scale and cognitive function scores, and CTP parameters were retrospectively analyzed.

**Results:**

The study cohort ultimately included data from 65 patients (60–68 years of age; median age, 66 years) who underwent 82 bypass surgeries. The patency rate of bridge arteries was 100% on intraoperative fluoroscopy and 95.0% (76/80) according to cerebral angiography at the last follow-up. The perioperative stroke rate was 1.54 % and the mortality rate was 3.08% in the 2nd year of follow-up. Compared with preoperative data, the mismatch volume of CTP was reduced (*P* < 0.001), and the Montreal Cognitive Assessment score significantly increased (*P* < 0.001) 2 years after bypass surgery. Forty patients in the cognitive improvement group had a higher educational level (*P* = 0.020), shorter course of disease (*P* = 0.041), shorter mean transit time (MTT) (*P* < 0.001), and shorter time to peak value (*P* = 0.015) on CTP, as determined by single-factor analysis before bypass, compared with those in the inactive group. Based on multivariate logistic regression analysis, a shorter preoperative MTT was an independent clinical factor for cognitive improvement after bypass (odds ratio 0.452 [95% confidence interval 0.082–0.760]; *P* = 0.003).

**Conclusion:**

EC–IC bypass surgery was safe and improved cognitive function in elderly patients diagnosed with ACMSO. Reversible cerebral perfusion function is one of the better prognoses, which needs to be confirmed in future study.

## 1 Introduction

Atherosclerotic carotid and/or middle cerebral artery (MCA) steno-occlusive (ACMSO) disease is a significant and increasingly common pathology among individuals of advanced age (Li et al., 2022; Cardenas et al., 2012) and plays a dominant role in the etiology of stroke and cognitive decline (Feigin et al., 2022; Sharrief and Grotta, 2019). Despite the lack of benefits of risk of stroke or death demonstrated by the Carotid and Middle Cerebral Artery Occlusion Surgery Study (CMOSS)-a recent pivotal clinical trial (Ma et al., 2023), CMOSS and the subsequent studies also indicated that a subset of patients with less severe hemodynamic insufficiency and/or mild complications insufficiency and may benefit from EC-IC bypass surgery, which requires further investigation (Sun et al., 2024; Oliveira et al., 2024).

Some experts believe that revascularization of the hypoperfused territory is still the most promising treatment strategy (Elder et al., 2024) and extracranial-intracranial (EC–IC) bypass surgery can improve clinical outcomes in patients with ACMSO through reversal of cerebral hemodynamic deficit (Cardenas et al., 2012; Komotar et al., 2009). Our previous studies revealed that asymptomatic patients with cerebral ischemia exhibited deficits in global cognition, memory and executive function (Shen et al., 2023); and also found a significant proportion of patients experienced relief of symptoms, specifically improving cognitive function after EC–IC bypass surgery (Du et al., 2023). As our opinion, the role of EC–IC bypass surgery for cognitive function has been neglected and should be fully evaluated, specifically for elderly patients with ACMSO.

As such, the present study is a retrospective approach through mainly cognitive function assessment and brain computed tomography perfusion (CTP) test before and after EC-IC bypass surgery to investigate the safety and cognitive outcomes of EC–IC bypass for the treatment of ACMSO in elderly patients.

## 2 Materials and methods

### 2.1 Clinical data

Data from patients diagnosed with ACMSO between January 2018 and January 2021 were retrospectively collected and reviewed. Eligibility criteria included the following: age, ≥60 years; presence of atherosclerotic severe stenosis and/or occlusion in internal carotid artery (ICA) or the MCA confirmed by digital subtraction angiography (DSA); mild cerebral ischemia symptoms with a National Institute of Health Stroke Scale (NIHSS) score of 1–4 points, and < 2 points on the Modified Rankin Scale (mRS); computed tomography (CT) perfusion (CTP) demonstrated hemodynamic insufficiency in the MCA territory; mild-moderate cognitive impairment before EC–IC bypass surgery; and fully understand the EC–IC bypass procedure and provided informed consent.

The exclusion criteria were as follows: Moyamoya disease features identified on DSA; Previous stenting or other endovascular interventions; atrial fibrillation on preoperative electrocardiography; Posterior circulation ischemia manifestations, including swallowing difficulties, ataxia, and dystonia; intracranial hemorrhage within 3 months or severe cerebral infarction within 1 month; Hypertension, diabetes, hyperlipidemia or other complications were poorly controlled; and loss to follow-up.

During the period of clinical study, the same number patients with similar baseline information who had just received conservative treatment were included in control group. Medical comorbidities, such as hypertension, diabetes mellitus, dyslipidemia, and coronary disease, were recorded after reviewing medical charts.

As our previous clinical research experience, the rates of stroke and death have no significant difference between bypass group and control group. The MoCA score was defined as the primary endpoint, of which the baseline score in control group and bypass group was about 20 points. According to our predictions, the mean score of control group had no change during follow-up, and the mean score of bypass group would increase from 20 points to 23 points and the standard deviation was 3 points during 2 years of follow-up. The formula was as follows: *n* = [(Z_α/2_+ Z_β/2_)/δσ]; Z: the critical value of the standard normal distribution; δ: The difference between the two groups was 3 points; σ: The standard deviation was 3 points. The test level was bilateral α= 0.05, the power was 80%. It is calculated that the minimum sample size of each group is 17 cases.

All study protocols were approved by the Institutional Review Board of Huadong Hospital (No. 20171205, Shanghai, China) and all individuals provided informed consent before participating in the study.

### 2.2 Surgical procedures

The surgical technique used for EC–IC bypass surgery was consistent with the authors' previous investigation (Du et al., 2023). A suitable recipient vessel, the cortical segment MCA (diameter ≥0.8 mm), was identified under a microscope. The superficial temporal artery (STA) was passed through the temporal muscle and anastomosed to the MCA. Bypass patency was confirmed intraoperatively using indocyanine green angiography.

### 2.3 Postoperative evaluation

Postoperative serial CT evaluations were performed on all patients immediately after surgery to rule out bleeding or infarction. If neurological deterioration was found, postoperative magnetic resonance imaging with diffusion-weighted imaging and T2-weighted imaging were performed, if necessary.

### 2.4 CTP imaging

CTP imaging acquisition and analysis methods were consistent with those used in the authors' earlier study (Du et al., 2023), which were typically performed preoperatively and 2 years after bypass surgery.

Thresholds for perfusion deficit and ischemic core detection were as follows. The threshold for identifying perfusion deficits was based on previously published criteria: Tmax > 6 s (Albers et al., 2018). A relative cerebral blood flow (rCBF) threshold < 30% was applied to quantify the ischemic core within the perfusion deficit area (Campbell et al., 2011). The CTP fitting images were shown on processing software (PerfusionGo V1.0, Shukun, Co. Ltd., China). Mismatch volume was defined as the difference between the perfusion deficit volume and the ischemic core volume. The region of interest measured 1.5 cm^3^ at the temporal area of MCA perfusion (paraventricular) in CTP fitting images, which was set on the left side of patients who underwent bilateral bypass surgery and the operative side for those who underwent unilateral bypass surgery.

### 2.5 Neurocognitive function score

All patients underwent neuropsychological tests preoperatively and 2 years after bypass surgery. Cognitive function(s) was assessed using the Chinese version of the MoCA (30 full scores) with 7 subsection items, including visual/executive (trail making, cube copy and clock draw), naming, attention (digit span, number, serial subtraction), language (repetition, fluency), abstraction (train, watch), delayed memory and orientation (day, month, year, week, place, city) (Du et al., 2024). An increase of ≥ 3 points on the MCoA after bypass surgery was defined as improvement and those patients were assigned to the improved group. An improvement of < 3 points or a decline was regarded to be ineffective, and these patients were assigned to the inactive group.

Patients were regularly assessed using the mRS and NIHSS scores at 6 months, and 1 and 2 years after bypass surgery. Cerebral angiography was performed 6 months after bypass surgery to confirm graft patency.

### 2.6 Statistical analysis

Data analysis was performed using SPSS version 23.0 (IBM Corporation, Armonk, NY, USA). Continuous variables were tested for normality using the Kolmogorov–Smirnov test. Normally distributed data are expressed as mean ± standard deviation, and the independent *t*-test was used for between-group comparisons. Non-normally distributed data are expressed as median (first quartile, third quartile) and between-group comparisons were performed using the Mann–Whitney U test. Categorical variables are expressed as frequency and were compared using the χ^2^ test. These variables were also compared between groups using χ^2^ or Fisher's exact tests. Univariate analysis identified potential risk factors cognitive improvement, which were entered into binary logistic regression analysis (advanced method) to determine the factors influencing post-bypass cognitive improvement. Differences with *P* < 0.05 were considered to be statistically significant.

## 3 Results

### 3.1 Overall characteristics

One hundred fifty patients were enrolled in this study in bypass surgery group, data from 65 patients (median age, 63 years), diagnosed with severe atherosclerotic cerebral steno-occlusive disease of the ICA or MCA, as verified by preoperative DSA, were included in the analysis. ICA occlusion was present in 35 patients and severe stenosis in 5 patients. occlusion and severe stenosis of the horizontal portion was found in 20 and 5 patients, respectively. main cerebral ischemia symptoms among the patients included: transient syncope (*n* = 34); numbness and/or weakness in the extremities (*n* = 15); and transient dysphonia (*n* = 17). Sixty-five elderly patients with similar clinical presentations were included in control group, there were no statistic differences between control group and bypass group in age, symptoms, comorbidity, severity of intracranial vascular stenosis, mRS, NIHSS and MoCA scores.

### 3.2 CTP imaging

**Figure 1:**
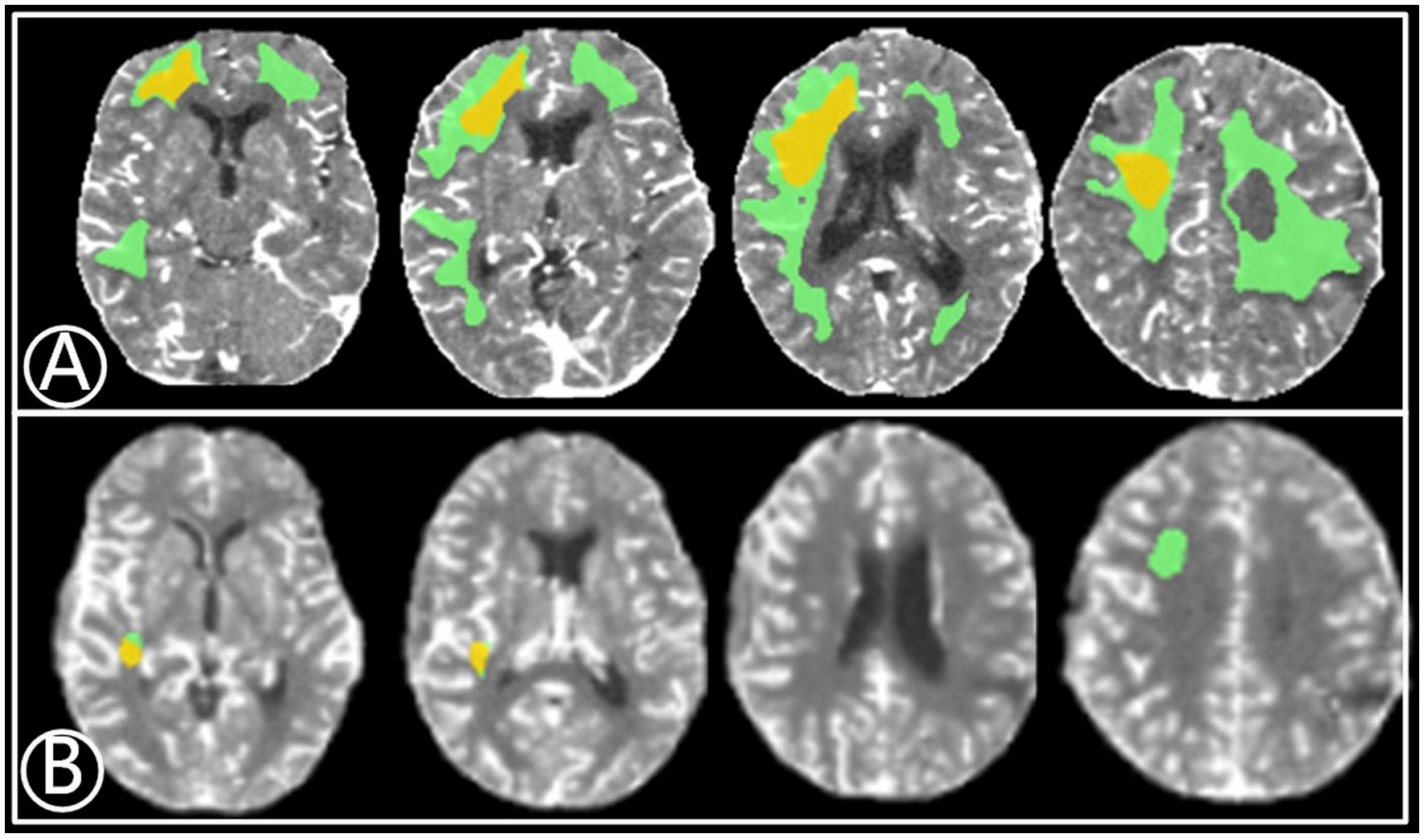
A 67-year-old male with a 2-month history of transient ischemic attacks. **(A)** Volume of relative cerebral blood flow (CBF) < 30% (yellow) was 15.6 mL^3^ before bypass and the mismatch area (green) was 41.2 mL^3^. **(B)** Eleven months after bypass, the volume of rCBF < 30% (red) and mismatch area (green) reduced to 1.9 mL^3^ and 3.2 mL^3^, respectively.

**Figure 2:**
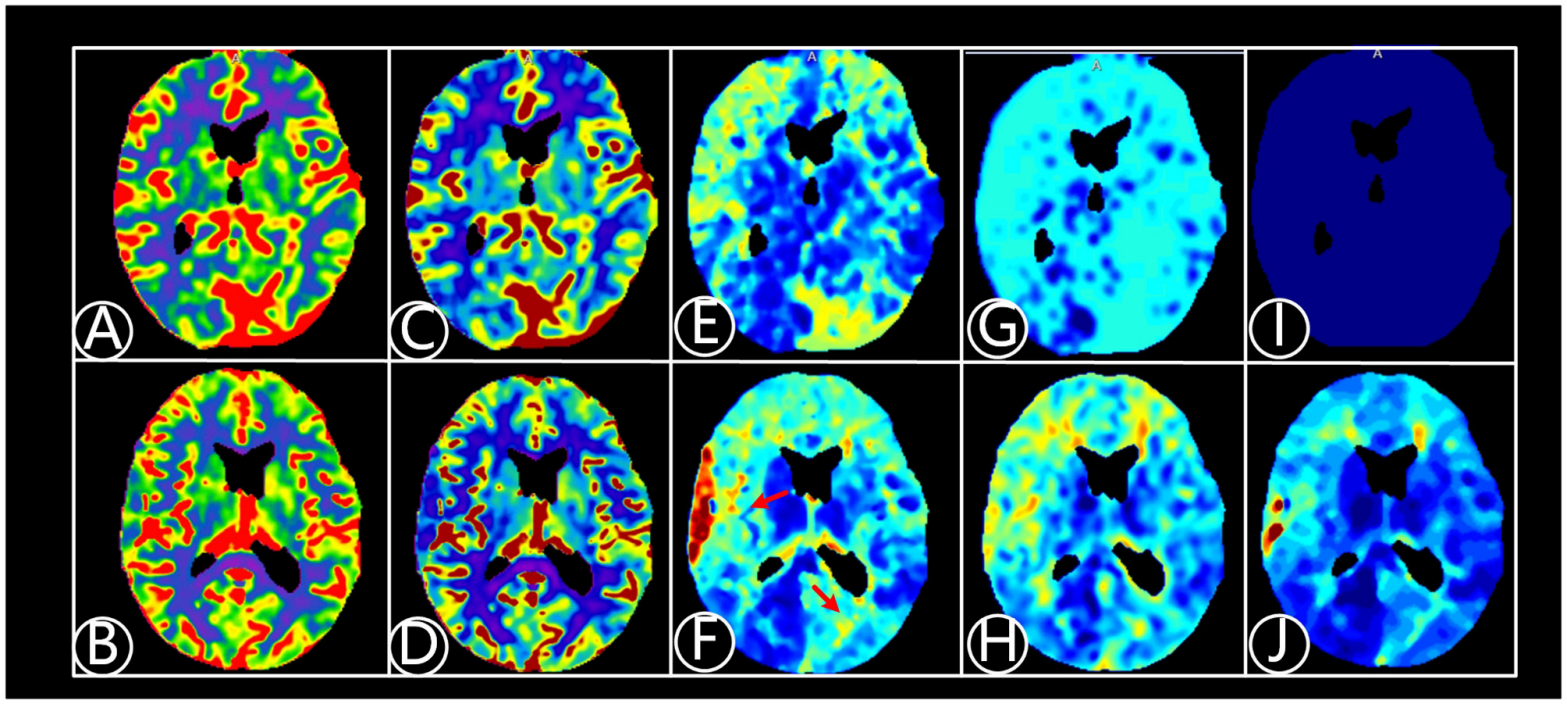
A 64-year-old patient suffered from dizziness for 5 years, being diagnosed with atherosclerotic arterial occlusion and underwent right EC-IC bypass surgery. CTP fitting images were shown before and 6 months after right EC-IC bypass surgery. **(A)** Fitting image of cerebral blood volume (CBV) before bypass surgery; **(B)** Fitting image of CBV after surgery; **(C)** Fitting image of cerebral blood flow (CBF) before surgery. **(D)** Fitting image of CBF after surgery; **(E)** Fitting image of mean transit time (MTT) before surgery; **(F)** Fitting image of MTT after surgery. Cerebral perfusion improves after surgery shown on red arrow area; **(G)** Fitting image of time to peak (TTP) before surgery; **(H)** Fitting image of TTP after surgery; **(I)** Fitting image of Tmax before surgery; **(J)** Fitting image of Tmax after bypass surgery.

In the bypass group, all patients exhibited abnormal cerebral perfusion and 61 of 65 had bilateral hemispheric impairment before bypass surgery. The unilateral cerebral hemisphere was divided into 5 brain regions: frontal, temporal, parietal, occipital, and basal ganglia, and 10 regions were present in the bilateral hemispheres. According to preoperative CTP imaging results, 2 patients had cerebral hypoperfusion in ≥8 regions, 50 patients had 5–7 hypoperfusion regions, and 13 had 3–4 hypoperfusion regions. Compared with the results before bypass and 2 years after, the mismatch volume significantly decreased from 36.3 ± 10.5 ml^3^ to 18.0 ± 6.4 ml^3^ (*P* < 0.001), as shown in Figure 1. MTT and TTP decreased from 6.7 ± 1.6 s and 4.3 ± 1.0 s to 5.5 ± 1.4 and 2.3 ± 1.1 s, respectively (*P* < 0.001). However, there were no statistical differences in cerebral blood volume (CBV), CBF and Tmax (Figure 2).

### 3.3 EC–IC bypass surgery

**Figure 3:**
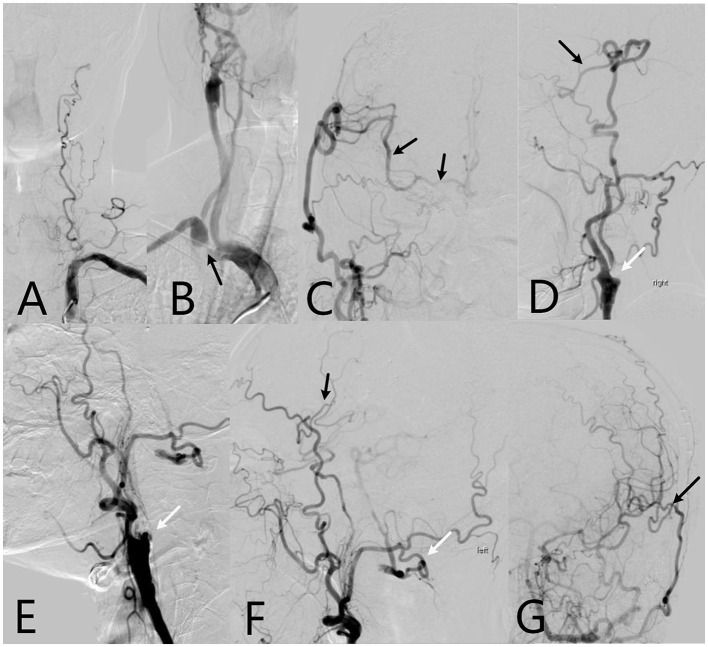
The 64-year-old patient's cerebral digital subtraction angiography (DSA) images. **(A)** Left vertebral artery is occlusive; **(B)** Right subclavian artery is severe stenosis (black arrow), and right internal carotid artery is occluded; **(C)** After 1 year of right EC-IC bypass surgery, Anteroposterior DSA image shows the right superficial temporal artery supplies the ipsilateral areas of the middle cerebral artery and bilateral of anterior cerebral artery through the anastomosis (black arrow); **(D)** Side position of DSA image shows new blood vessels in brain (black arrow), right internal carotid artery is still occluded (white arrow); **(E)** Left internal carotid artery is occluded. **(F)** After 7 months of left EC-IC bypass surgery, side position of DSA shows left superficial temporal artery communicates with the ipsilateral areas of the middle cerebral artery through the anastomosis (black arrow). The left occipital artery branch which supplies the left vertebral artery is protected well (white arrow). **(G)** Anteroposterior DSA image shows the EC-IC anastomosis is vibrant (black arrow).

Of 65 patients with ACMS enrolled in bypass surgery group, 17 patients with bilateral ACMSO underwent bilateral EC–IC bypass surgeries and 48 patients received unilateral surgery, resulting in 82 bypass surgeries in this cohort. All 82 STA-MCA anastomoses were unobstructed on intraoperative indocyanine green video angiography. Follow-up cerebral angiography revealed that 79 (96.3%) of the bypass grafts had maintained patency from 6 months to 4 years after EC–IC bypass surgery (Figure 3).

### 3.4 Neurological and cognitive function assessment

#### 3.4.1 mRS and NHISS

In surgery group, 32 patients experienced symptom relief during follow-up. mRS scores among the patients before bypass surgery were distributed as follows: 0 points (*n* = 28, 43.1%); 1 point (*n* = 34, 52.3%); 2 points (*n* = 3, 4.6%). The mean NHISS score at the preoperative assessment was 2.32 ± 0.92 and 2.28 ± 1.07 at 2 years after bypass surgery (*T* = 0.352, *P* = 0.726). There were also no statistical differences between preoperative score and all time points after bypass surgery. At the final follow-up 2 years after bypass, 41 patients experienced symptom relief, 5 deteriorated, and the remainder experienced no change based on the mRS, NHISS, and inquiries.

#### 3.4.2 Cognitive assessment scale

The control patients' mRS, NIHSS and MoCA assessment scores had no statistical changes during 2 years of follow-up. In surgery group, MoCA scores significantly increased from 20.6 ± 3.0 pre-bypass to 22.8 ± 3.8 at 2 years postoperatively and the abilities at subitems of visual/executive, language, delayed memory improved statistically (Table 1). Except 2 patients in bypass surgery group died during follow-up, rest of 63 patients completed all assessments. Forty-three patients were assigned to the improved group and 20 to the inactive group (Table 2). Single-factor analysis revealed that patients who improved comprised a greater proportion of higher education (senior high school or above, *P* = 0.020), shorter disease duration (*P* = 0.041), shorter MTT (*P* < 0.001), and TTP (*P* = 0.015). Multivariate logistic regression analysis identified preoperative shorter MTT as an independent clinical factor for cognitive enhancement after bypass (odds ratio 0.452 [95% confidence interval 0.082–0.760]; *P* = 0.003) (Table 3).

**Table 1:** The MoCA and subitems score of control group and the patients before and after extracranial–intracranial (EC–IC) bypass surgery.

**Item (score)**	**Control (*n =* 65)**	**Pre-bypass (*n =* 63)**	**Post-bypass (*n =* 63)**	**Test value**	***P*-value**
Total MoCA (30)	20.0 ± 3.4	20.6 ± 3.0	22.8 ± 3.8	−14.084^a^	< 0.001
**Subitems**					
Visual/executive (5)	3 (2.4)	4 (3.4)	4 (5.4)	−4.433^b^	< 0.001
Naming (3)	2 (2.2)	2 (2.2)	2 (2.2)	−0.577^b^	0.564
Attention (6)	4 (3.4)	4 (2.4)	4 (3.5)	−1.33^b^	0.184
Language (3)	2 (2.2)	2 (2.2)	3 (2.3)	−4.419^b^	< 0.001
Abstraction (2)	2 (2.2)	2 (2.2)	2 (2.2)	−1.414^b^	0.157
Delayed memory (5)	2 (1.3)	2 (2.2)	3 (2.3)	−2.521^b^	0.012
Orientation (6)	5 (5.6)	5 (4.6)	6 (5.6)	−3.741^b^	< 0.001

ACMSO, Atherosclerotic internal carotid artery and/or middle cerebral artery steno-occlusive; MoCA, Montreal Cognitive Assessment; EC-IC, extracranial–intracranial. The Mann–Whitney U test was compared to assessment scores before and after EC-IC bypass surgery. ^a^T value; ^b^Z value.

**Table 2:** Single-factor analysis of cognitive function status in elderly patients with ACMSO after EC-IC bypass surgery.

**Items**	**Total (*n =* 65)**	**Inactive group (*n =* 20)**	**Improved group (*n =* 43)**	**Test value**	***P*-value**
Age	63 (61, 65)	64.5 (62, 66.8)	63 (61, 65)	−0.435^c^	0.663
Male (*n*, %)	29	13 (52.0%)	26 (60.5%)	1.038^b^	0.298
low education level	36	10 (40%)	26 (60.5%)	3.891^b^	0.041
Course of disease	7 (3, 13.5)	9.5 (7.3, 17.3)	6 (3, 12.5)	−2.326^c^	0.020
**Anterior circulation injured**					
Bilateral impaired	25	11 (44.0%)	14 (32.5%)	0.526^b^	0.468
Dominant hemisphere impaired	37	16 (64.0%)	21 (48.8%)	0.830^b^	0.362
Posterior circulation injured	13	4 (16.0%)	9 (22.5%)	0.406^b^	0.524
**Main clinical symptoms**					
Transient syncope	44	12	26		
Numbness and weakness in extremities	17	6	15		
Speech disorder	4	2	2		
**Vascular injury related factors**					
Hypertension	21	6 (24.0%)	15 (34.9%)	1.282^b^	0.258
Diabetes	16	8 (32.0%)	8 (20.9%)	1.194^b^	0.275
Hyperlipidemia	23	8 (32%)	15 (34.8%)	0.264^b^	0.625
Smoking	9	4 (16.0%)	5 (11.6%)	0.158^b^	0.691
Drinking alcohol	14	6 (24.0%)	8 (18.6%)	0.146^b^	0.703
**Laboratory examination**					
Red blood cell count (10^12^/L)	4.6 ± 0.94	4.4 ± 0.79	4.7 ± 1.0	−0.118^a^	0.268
White blood cell count (10^9^/L)	9.5 (7.9, 11.1)	9.4 (7.3, 10.8)	9.5 (8.2, 11.3)	−7.151^c^	0.475
Neutrophil count (10^9^/L)	7.2 ± 2.13	6.7 ± 2.0	7.5 ± 2.2	0.842 ^a^	0.1308
Serum albumin (10^9^/L)	43.4 ± 5.3	43.3 ± 4.5	43.5 ± 5.8	0.002 ^a^	0.984
Blood platelet count (10^9^/L)	208.9 (133.6, 280.4)	179.2 (121.5, 276.2)	210.9 (147.3, 286.1)	−0.395 ^c^	0.338
D–dimer (mg/L)	1.7 ± 1.1	1.9 ± 1.1	1.6 ± 1.01	−0.799	0.427
**CTP test**					
CBF	33.3 ± 7.2	35.3 ± 6.5	32.0 ± 7.5	1.806^a^	0.072
CBV	2.10 (1.78, 2.29)	2.11 (1.73, 2.34)	2.09 (1.75, 2.33)	−0.525	0.591
MTT	6.7 ± 1.6	7.6 ± 1.9	6.2 ± 1.2	3.742^a^	< 0.001
TTP	4.3 ± 1.0	4.7 ± 0.9	4.1 ± 1.1	2.498^a^	0.015
Tmax	5.2 (3.34, 7.40)	5.3 (4.3, 6.4)	4.5 (3.2, 7.7)	−0.829	0.406

^a^T value; ^b^χ^2^ value; ^c^Z value. EC–IC, Extracranial–intracranial; ACMSO, Atherosclerotic internal carotid artery and/or middle cerebral artery steno-occlusive; CBF, cerebral blood flow; CBV, cerebral blood volume; MTT, Mean transit time; TTP, Time to peak.

**Table 3:** Multivariate logistic regression analysis of cognitive improvement in elderly patients with ACMSO after bypass surgery (*n* = 63).

	**Partial regression coefficient**	**STANDARD error**	**Wald χ^2^ value**	**OR value**	**95% CI**	***P* value**
High educational level	−1.244	3.763	3.763	0.288	0.082–1.013	0.052
Short course of disease	−0.058	3.256	3.256	0.944	0.082–1.005	0.071
Short MTT	−0.794	8.972	8.972	0.452	0.082–0.760	0.003
Short TTP	−0.602	3.242	3.242	0.548	0.082–1.055	0.072

ACMSO, Atherosclerotic internal carotid artery and/or middle cerebral artery steno-occlusive; MTT, Mean transit time; TTP, Time to peak.

### 3.5 Adverse events

In bypass series, one (1.54%) patient experienced severe stroke 3 days postoperatively and passed away 1 month after the bypass surgery, while another died of heart failure 13 months after bypass surgery. The total mortality rate was 3.1% in the 2nd year of follow-up, and the other two patients (3.1%) had moderate to serious stroke during follow-up. In control group, two patients had moderate to severe stroke and one patient died because of pulmonary infection, and the morbidity and mortality was 3.1% and 1.5%, respectively, and there was no statistic difference between control group and bypass group in adverse event rates.

## 4 Discussion

EC–IC bypass surgery has traditionally been the surgical method of choice for treating occlusive diseases affecting the extracranial and intracranial cerebral vessels, aneurysms, carotid-cavernous fistulas, cerebral vasospasms, acute cerebral ischemia, and Moyamoya disease (Reddy et al., 2022). However, whether this surgical intervention can mitigate risks of stroke and improve neurological function, especially for cognition, memory, and intellect in elderly patients with ACMSO remains controversial (Ma et al., 2023; Wessels et al., 2021). In this study, it shows EC–IC bypass surgery is safe treatment for elderly patients with ACMSO, most of them (66.2%) can receive cognitive function improvement after surgery. Furthermore, a shorter preoperative MTT score was an independent clinical factor associated with cognitive enhancement following bypass surgery.

### 4.1 Safety of EC–IC bypass in the elderly

In this cohort, the incidence of stroke and mortality rates were 3.1% and 1.5%, which was not found to be inferior to medical interventions. As the discouraging result of the CMOSS Randomized Clinical Trial, bypass surgery combined with medical therapy did not significantly change the risk of stroke or death for the patients with symptomatic ACMSO during follow-up of 2 years, compared with simply medical therapy (Ma et al., 2023). Another multicenter, prospective nonrandomized study showed similar clinical outcomes that carotid artery stent placement and carotid endarterectomy procedures did not seem to reduce the risk of stroke at follow-up compared with medical treatment and the two kinds of carotid near-occlusion revascularization were associated with high rates of failure and periprocedural complications (Garcia-Pastor et al., 2022). Risk factors for systemic atherosclerosis have been identified, including age, male sex, family history, smoking, hypertension, hyperlipidemia, a sedentary lifestyle, and a high-fat diet (Qaja et al., 2025). The mechanism underlying cerebral stroke often involves embolization of intracranial vascular plaques with hemodynamic compromise due to stenosis, potentially leading to a persistent detrimental cycle (Choi et al., 2019). Based on our postoperative CTP and DSA results, STA–MCA bypass can establish adequate cerebral perfusion by creating a new circulation pathway from the EC artery to the IC artery (Du et al., 2023). Possible mechanisms include the recovery of CBF perfusion and improvement of metabolic function over time (Turpin et al., 2023). It is noteworthy that patients experiencing focal small cerebral ischemia or mild transient ischemic attacks potentially derive greater benefits from bypass surgery than those with serious stroke (Sebök et al., 2024).

In Sun' recent study, the patients with ACMSO who had higher level of plasmic triglyceride-glucose index were associated with poorer outcomes after EC-IC bypass (Sun et al., 2024). As our long-term clinical practice, strict and scientific management of internal medicine diseases, such as hypertension, diabetes and hyperlipidemia and healthy lifestyle preoperatively for at least 3 months, are helpful for reducing preoperative complications (Reiff et al., 2022). In this cohort, more severe hemodynamic insufficiency or stroke (longer MMT) may mean a higher incidence of postoperative stroke (Ma et al., 2023), which is associated with poor cognitive outcomes, and we suggest that shorter MMT (< 7 s) and without CBV and CBF impairment in ipsilateral brain can be surgical threshold, which should be explored in future study.

In the bypass group of this study, all patients exhibited abnormal cerebral perfusion and 93.8% (61/65) had bilateral hemispheric impairment before bypass surgery and cerebral hemodynamic compromise was confirmed through CTP imaging by drawing a clear outline of the abnormal mismatch volume. Chen et al. also reported that EC–IC bypass surgery can effectively irrigate the ischemic zone (Chen and Tu, 2022). In this study, after 2 years of bypass, the elderly patients' mismatch volume, and values of MTT and TTP all showed significant improvements. As previous reports showed that CTP could serve as an effective tool for diagnosing and assessing changes in cerebral perfusion parameters in patients with severe intracranial steno-occlusive disease, and be helpful in the selection of treatment strategies and the evaluation of prognosis (Qiao et al., 2022). Based on our multivariate logistic regression result, preoperative shorter MTT is an independent benefited factor for cognitive enhancement after bypass. We also suggest CTP can be a sensitive functional imaging tool for testing cerebral microcirculation, and assessing the changes in cerebral perfusion during follow-up for both those patients with bypass and conservative treatments and future studies may focus on identifying those patients with potential benefits or pinpointing a more precise location for anastomosis in hypoperfusion areas (Ding et al., 2025; Klug et al., 2021).

### 4.2 EC–IC bypass surgery improves cognitive function

Hemodynamic compromise may give rise to classic stroke or transient cerebral ischemia, but may also be less predictable and atypical, such as generalized fatigue, cognitive disorder, limb shaking, headache, and syncope (Qaja et al., 2024). According to our previous study, it was demonstrated that asymptomatic moyamoya adults also suffered from moderate to severe cognitive deficits, especially in memory and executive function (Shen et al., 2023). Successful unilateral EC–IC bypass did not adversely affect postoperative cognitive function (Inoue et al., 2016a) and but also improved some cognitive domains, including verbal memory, visual memory, executive function, attention, and psychomotor speed by correcting cerebral hemodynamics (Li et al., 2018; Doherty et al., 2021). In this cohort, MoCA score in bypass surgery group had significantly increased, especially at subitems of visual/executive function, language, delayed memory. On the one hand, the improvement of cerebral blood perfusion and relief of symptoms associated with cerebral ischemia may be the most important reason; on the other hand, postoperative rehabilitation training and hyperbaric oxygen therapy may also provide positive support in some ways. Possibly, there may be a threshold for hemodynamic impairment that affects the reversibility of cognitive function after an EC–IC bypass. For example, patients with severe hemodynamic impairment and serious stroke may experience substantial neuronal injury or further deterioration and cognitive function may not be reversible even after hemodynamic correction through a successful bypass (Inoue et al., 2016b). However, MoCA as a tool for cognition screen cannot evaluate the cognitive change comprehensively. In the future, a more systematic cognitive assessments should be practiced, specially focusing on executive function, language, memory function, based on the results of this study. Our results of single factor and multi-factor analysis that shorter disease duration, and only shorter MTT and TTP, and without CBV and CBF impairments in CTP test can receive better cognitive improvements after EC–IC bypass surgery, indicating those patients with mild cerebral ischemia can have better prognosis. Li et al. (2018) also showed that patients with a defined compensatory stage of cerebral ischemia with normal CBF, normal or increased CBV, and normal oxygen extraction fraction in the CTP test showed a better prognosis following EI–CI bypass surgery. Horfman found a high coefficient of variation of MTT in 3 weeks was a poor predictive factor for those patients with aneurysmal subarachnoid hemorrhage (Hofmann et al., 2021). In conclusion, the restoration of the hemodynamic state by surgical revascularization could theoretically contribute to postoperative cognitive improvement in elderly patients diagnosed with mid-ACMSO.

### 4.3 Limitations

The present study had some limitations, the first of which were its retrospective, single-center design, brief follow-up, and small sample size. In the future, prospective randomized controlled trials involving multiple centers are needed to further verify the efficacy of EC–IC bypass in elderly patients with ACMSO and to investigate the underlying mechanisms of cognitive improvement.

## 5 Conclusion

EC–IC bypass surgery was safe and effective in elderly patients with ACMSO. Increased cerebral perfusion after bypass surgery significantly improved cognitive function. The role of MMT in CTP test before bypass should be further explored in future.

## Data Availability

The raw data supporting the conclusions of this article will be made available by the authors, without undue reservation.
